# Race, Poverty, and Foster Care Placement in the United States: Longitudinal and Cross-Sectional Perspectives

**DOI:** 10.3390/ijerph20166572

**Published:** 2023-08-13

**Authors:** Fred Wulczyn, Xiaomeng Zhou, Jamie McClanahan, Scott Huhr, Kristen Hislop, Forrest Moore, Emily Rhodes

**Affiliations:** Center for State Child Welfare Data, Chapin Hall Center for Children, University of Chicago, Chicago, IL 60637, USA; jmcclanahan@chapinhall.org (J.M.); shuhr@chapinhall.org (S.H.); khislop@chapinhall.org (K.H.); fmoore@chapinhall.org (F.M.);

**Keywords:** child poverty, foster care placement, racial disparity, United States

## Abstract

Although the connections between race, poverty, and foster care placement seem obvious, the link has not in fact been studied extensively. To address this gap, we view poverty and placement through longitudinal and cross-sectional lenses to more accurately capture how *changes* in poverty rates relate to *changes* in placement frequency. The longitudinal study examines the relationship between poverty rate changes and changes in the placement of Black and White children between 2000 and 2015. The cross-sectional study extends the longitudinal analysis by using a richer measure of socio-ecological diversity and more recent foster care data. Using Poisson regression models, we assess the extent to which changes in race-differentiated child poverty rates are correlated with Black and White child placement frequencies and placement disparities. Regardless of whether one looks longitudinally or cross-sectionally, we find that Black children are placed in foster care more often than White children. Higher White child poverty rates are associated with substantially reduced placement differences; however, higher Black child poverty rates are associated with relatively small changes in placement disparity. Black and White child placement rates are more similar in counties with the fewest socio-ecological assets.

## 1. Introduction

The right to raise one’s children is fundamental to U.S. social policy [1], so foster care placement frequency is an essential barometer of the human, social, economic, and political dynamics that affect this most basic human right [2]. In this paper, we examine the relationship between race, child poverty, and the frequency of placement away from home and into foster care. Although the connections between race, poverty, and placement seem obvious on their surface, the link has not been studied extensively.

To address this gap, we view poverty and placement through longitudinal and cross-sectional lenses to more accurately assess how *changes* in poverty rates relate to *changes* in placement frequency. For the longitudinal perspective, we use county-level race-specific admission counts for three census years (2000, 2010, and 2015) together with county-level, race-specific measures of poverty to observe how poverty rate changes affect placement frequency. The cross-sectional study is based on a database that uses a more contemporary sample of foster care admissions (2017–2019). We use the cross-sectional study to expand how we think about socio-economic circumstances. Rather than rely solely on measures of poverty, we create an index of social disadvantage using multiple social indicators assembled from the census and other county data sources [3]. The results add clarity to what the longitudinal study shows.

Substantively, we are interested in showing how the poverty rate changes between 2000 and 2015 influenced placement frequency and placement disparity. Our focus is on Black child poverty, White child poverty, Black child placement, and White child placement. With these data, we measure the average placement rate for each group alongside the poverty rate for each group. After adjusting for the size of the underlying population, we then measure the change in placement frequency for each group as a response to the changes in poverty. The poverty, race, and placement relationship revealed is a powerful but somewhat contradictory narrative of how economic hardship measured at the population level affects the use of foster care.

## 2. Literature Review

Regarding the use of foster care measured as a placement rate (i.e., the number of children placed per unit population and unit of time), the research is rather fragmented, with surprisingly little attention paid to placement frequency [4]. As a precursor to placement, child maltreatment/differential investigation rates receive considerable attention in the literature [5,6,7,8]. In this literature, foster care is treated as one of several options caseworkers may choose when managing a child protection case. However, when placement is conditional on a maltreatment report, the measure used is a probability rather than a frequency. Without an answer to the frequency question, we cannot say whether placement among Black children is more common than it is among White children or whether placement in foster care is becoming more or less common as social conditions surrounding families change.

On the other hand, there is a robust literature that describes what happens to children once they are placed in foster care (i.e., their outcomes). In this literature, the outcomes tend to be measured in terms of permanency and/or well-being [9,10,11,12,13], but again these outcomes do not address how often placement happens or the reasons why.

The literature that examines placement frequency is rather sparse by comparison. Several recent studies have focused on foster care and substance use as a cause of foster care admissions, but these works do not touch specifically on race and disparity [14,15,16,17]. There is research that explores cumulative placement rates, but that work is focused on individual-level probabilities over the life course [18,19]. When foster care placement rates (e.g., the rate per 1000 children) are the subject of research, the analysis has tended to focus on poverty and other measures of social disadvantage [3,20,21,22] as causes. Myers and colleagues [23] carefully review the distinction between disparity and disproportionality with reference to foster care placement, but they do not link the measures to poverty or changes in poverty. This gap in the literature needs to be filled if we hope to do something about the reasons why admissions to foster care are more common among Black children than White children.

As for the focus on poverty at the county level, we see poverty rates measured at the ecological level as a marker for what might be called the headwinds that make holding a family together more difficult. For example, where poverty rates are elevated, the people who live in those counties have less access to health care, less daycare, poorer schools, and so on [24]. Poverty also undermines collective efficacy/social capital which affects child monitoring [25,26,27,28]. Poverty is also associated with differences in parenting styles [29]. There is, as well, extensive literature that links economic disadvantage to maltreatment reporting as a precursor to foster care placement [8,30,31,32,33,34,35]. In sum, raising children amid impoverished conditions is harder for everyone. Though there are other factors involved, the baseline assumption is that foster care placement rates, as a measure of placement frequency, will be higher in counties with elevated poverty rates because being a successful parent there, no matter how that term is defined culturally, is more difficult unless there are countervailing influences that blunt poverty’s coercive effects on parents trying to hold their families together.

Whether the county is a useful unit of analysis is a reasonable question. In the foster care context, courts play a role in whether a young person will go into foster care, and the courts are often organized at the county level. It is reasonable to assume, therefore, that county-level administrative practices influence placement frequency. Additionally, in nine states, child welfare services are administered at the county level. More generally, in their paper on the effects of neighborhoods on intergenerational mobility, Chetty [36] note that counties are much larger than neighborhoods. However, they also note that the variance of place effects at the county level is a lower bound for the total variance for neighborhood effects which includes local variation [37].

With that said, from our perspective, the analysis presented is not meant to close the door to what is a very complicated question. Placement rates vary at the county level, as do county poverty rates. The county-level covariation we observe fills but does not complete the poverty/placement narrative. The differences we find have to be confronted with more penetrating research that acknowledges the various levels of analysis with theoretical relevance: child, family, worker, community resource capacity, and so on. The work presented here is but a first step in a more comprehensive analysis.

## 3. Research Questions

To describe the relationship between poverty, race, and foster care placement, we answer a series of basic but interrelated questions. The first set of questions focuses on poverty rates and placement rates for both Black and White children. Poverty rates and poverty rate variation between counties differ for Black and White children, so any reckoning of how poverty and placement are related has to take these basic differences into account. Four questions frame the answer:

▪ What is the average child poverty rate for Black children and White children?
▪ What is the disparity rate when using the poverty rates?
▪ What is the average rate of placement for Black children and White children?
▪ What is the disparity ratio based on those rates?

A second set of questions considers poverty rate changes and placement changes over time. The longitudinal view brings us closer to understanding how poverty and placement might be related [4] because it measures how *changes* in one relate to *changes* in the other. We know that poverty rates changed for both Black and White children but at different rates depending on whether the counties being considered are urban or rural in character [38,39]. We also know that any urbanicity effects fall more squarely on Black children whereas rural effects fall more squarely on White children because of geographic segregation [40,41,42,43,44].

▪ For Black children and White children, how have race-specific poverty rates changed?
▪ How have race-specific poverty rate changes affected race-specific placement changes, after adjusting for population size?
▪ How have the poverty rate changes affected placement disparities?

The last question we ask addresses the concern that poverty, as a measure of population health, may not be adequate. In particular, the literature on collective efficacy and social capital suggests that poverty measures miss the social cohesion that serves as a protective factor within communities [25,45,46,47,48]. To address this potential shortcoming of our poverty measure, we devise a more nuanced index of social disadvantage that combines measures of income, family structure, and general well-being (mortality rates, housing problems, and so on) to better capture, albeit imperfectly, the unmeasured protective aspects of community life. Details of how the index was constructed are found in the Appendix A.

▪ Given poverty’s effect on race-specific placement frequency, does a more nuanced measure of social disadvantage add detail to the emerging placement disparity narrative?

## 4. Methods

### 4.1. Sample Data

The foster care admission data comes from the Multistate Foster Care Data Archive (FCDA). The count of admissions includes children under the age of 18 admitted for reasons of abuse, neglect, or dependency (which includes situations wherein the child has no parent, guardian, or custodian responsible for care and supervision). To the extent they are counted as part of a state’s foster care population, we excluded cases of juvenile delinquency. The admission count uses only first-ever admissions (i.e., inception cohorts).

From the FCDA, for the two studies, we selected the largest group of states with reliable admission counts for 2000, 2010, 2015, 2017, 2018, and 2019. The first three years were used for the longitudinal study; the last three years were used for the cross-sectional study. A total of 18 states from diverse parts of the United States were selected.

For the foster care placement rate, we obtained county-level Black and White child population estimates from the 2000 and 2010 U.S. Census. For 2015, the population estimates come from the five-year American Community Survey (ACS) estimates. For the cross-sectional study, we used the total number of first foster care placements during 2017–2019. To compensate, we multiplied the county child population by three to obtain a properly scaled denominator. The benefit of joining three years together in this way comes from how it stabilizes placement counts and rate estimates for small counties. For counting both Black and White child foster care placements, we focus on all non-Hispanic Black children and all non-Hispanic White children. For the Black and White child population estimates, the same rules were applied.

Once the data were assembled, we adjusted the final sample of counties. Counties with missing values for the Black or White population data were excluded from the sample. As a result, 892, 1021, and 835 counties for 2000, 2010, and 2015, respectively, were included in the longitudinal study and 965 counties were included in the cross-sectional study. Details of the study samples can be found in Table A1, Table A2, Table A3 and Table A4.

### 4.2. Variables

Dependent variable: The dependent variable is the number of children placed in foster care for the first time in the aforementioned years (i.e., the frequency). Each county has two placement frequencies: the number of White children placed and the number of Black children placed. Because these two frequencies alone do not have much meaning, we use the number of White children and Black children living in each county as an exposure variable in a Poisson count model [3,49,50]. The exposure variable adjusts the placement frequency for the size of the Black and White child population, respectively.

Independent variables: To describe the relationship between race, poverty, and placement we include three independent variables in our analysis. For the longitudinal analysis, we focus on race-specific child poverty rates and urbanicity; for the cross-sectional study, we add an index of social disadvantage that is similar to one used previously [3]. 

The poverty rates for Black and White children were assembled from the census data as described previously. The urban-rural classification scheme was borrowed from the National Center for Health Statistics (NCHS). The NCHS urban-rural classification uses six categories in descending order on the urban/rural continuum: large central counites, large fringe counties, medium metro counties, small metro counties, micropolitan counties, and non-core counties. To simplify, the original six categories were further grouped into two categories for the multivariate analysis, with the first two categories combined into one and the last four categories grouped into one (i.e., urban counties and non-urban counties).

In addition to poverty and urbanicity, the cross-sectional study includes an index of social disadvantage (see Table A5). We constructed the index using 20 publicly available county-level indicators in three domains (poverty, family structure, and general social indicators) by measuring each county’s deviation from each indicator’s mean and then summarizing those deviations across all 20 indicators. In the resulting index, the counties were grouped into seven categories, where counties in category 7 were those with low social disadvantage and counties in category 1 were those where parents face far more challenging circumstances when raising children (high social disadvantage).

## 5. Analysis

In the data file used for the analysis, each county has two sets of observations: (1) a record that contains the count of Black child foster care admissions plus the count of Black children in the general population and (2) a record that contains the count of White child foster care admissions plus the count of White children in the general population. We use a binary variable in our analysis to differentiate the observations (the records) of Black children from the observations (the records) of White children. For interpretative clarity, we refer to this binary variable as Disparity in the multivariate models. We use this term because the coefficient, as detailed below, measures the Black child/White child placement rate difference (i.e., disparity).

The analysis begins with the log link. In this context, the Poisson model with the log link is:

(1) $$E\left(Y_{ij}|\lambda_{ij}\right)=L_{ij}\lambda_{ij}\;\mathrm{and}\;\log\left(\lambda_{ij}\right)=\beta_{j}D_{ij}=\beta_{0j}+\beta_{1j}D_{ij},\;i\in \left\{0\;or\;1\right\},\;\;j\in \left\{1,2,\ldots ,J\right\}$$

Under the Poisson distribution, the expected value of Y*_ij_* (the number of placements), given the event rate (λ*_ij_*), is the event rate (λ*_ij_*) multiplied by the number of children living in the population (L*_ij_*). Recalling that each county has two observations, then if *i* = 1, the results pertain to the count of Black child placements and the count of Black children in county *j*, and the parameter Y*_ij_* is the number of Black placements. If *i* = 0, then the results pertain to the count of White child placements and the count of White children in county *j*, and the parameter Y*_ij_* is the number of White placements. With this file structure, the Poisson regression model accommodates both the Black child placement count and the White child placement count together with their respective population counts: log(L*_ij_* λ*_ij_* ) = log(L*_ij_*) + log(λ*_ij_*) = log(L*_ij_*) + β*_j_*D*_ij_*. Now the variable (L*_ij_*) is included on the right side of the equation as an exposure variable (i.e., the number of children in the population). The term D*_ij_* indicates either the White or Black placement count in county *j*, depending on whether the parameter is set to 0 or 1. 

To carry out the longitudinal analysis, we used this Poisson regression with a value-added specification that captures the *change in admission rates* and *the change in child poverty rates* over time. This is also referred to as a dynamic model. The dynamic model has two advantages over more conventional approaches when the data are structured correctly. First, the dynamic model incorporates the change in one variable and its effect on a second variable into the parameter estimates. This is a more direct measure of the change process. Second, by including the prior admissions with a population size adjustment as a covariate, it absorbs incoming admission differences and other unobserved factors that are not easy to capture otherwise. The simplest form of the dynamic model can be expressed as shown in Equation (2).

(2) $$Y_{it}=\beta \;Y_{it-1}\;\;i\in \left\{1\;or\;2\right\},t\in \left\{1\;or\;2\right\}$$

We have three-time points for county-level admissions and child poverty: 2000, 2010, and 2015. According to Equation (3), the three data points form two segments or cohorts—one from 2000 to 2010 and the other from 2010 to 2015. Both admission and child poverty were measured by race along with the change from 2000 to 2010 and from 2010 to 2015. Y*_it_* refers to the admissions in time *t* and Y*_it−_*_1_ refers to the admissions at the previous time *t−*1. The inclusion of the lagged outcome, Y*_it−_*_1_, makes it a dynamic model. The relationship of Y*_it−_*_1_ with Y*_it_* is expressed by the parameter β. Incorporating a dynamic model, the mixed or combined model becomes:

Log(λ*_ijt_*) = β_00_ + β_10_D*_ijt_* + β_1_Y*_ijt−_*_1_ + β_2_P*_ijt_* + β_01_ C*_j_* + β_02_ C_*jt−*1_ + β_11_ D*_ijt_* C*_jt−_*_1_+ β_03_ (C*_jt_* − C*_jt−_*_1_) + β_13_ D*_ijt_* (C*_jt_* − C*_jt−_*_1_) + Σα*_j_* + γ_0_*_j_* + γ_1_*_j_* D*_ijt_* (3)

The overall model intercept, β_00_, refers to the average admission rate (i.e., the event rate) in the population when all the covariate values are zero. β_1_ represents the coefficient for prior admissions. D*_ijt_* refers to Black population counts (*i* = 1) or White child population counts (*i* = 0) for county *j* at time *t*. This is the binary variable that indicates whether the outcome is for Black children or White children. This means that β_10_ measures the difference between the Black and White child placement rates. In multivariate model findings, β_10_ is labeled Disparity. The cohort-specific binary variable P*_ijt_* characterizes the unique dynamics between two cohorts, and β_2_ measures the impact of P*_ijt_*. For Cohort 1, *t* is 2010 and *t−*1 is 2000, and for Cohort 2, *t* is 2015 and *t−*1 is 2010. Σα*_j_* is a set of binary variables that represent individual state-specific intercepts. Counties are nested within a state and the variation between states is captured by the state-fixed effects, but we do not present individual state effects in the findings. We also ran county random coefficients models without state-fixed effects and found results that are consistent with the state-fixed effects models presented here.

Variable C*_jt−_*_1_ is a time-varying, race-specific county poverty rate for county *j* at time *t−*1, which may change from one measurement occasion to another. Because the Black and White child poverty rates may have different effects on the number of Black and White child placements, the interaction D*_ijt_*C*_jt-_*_1_ measures the differential impact of C*_jt−_*_1_ depending on D*_ijt_*. We measure changes in Black and White child poverty rates from time *t* to time *t−*1 (C*_jt_* − C*_jt−_*_1_) to investigate how race-specific poverty rate changes affect placement rates (β_03_) and the level of disparity (β_13_). The time-invariant county variable C*_j_* for the longitudinal study is urbanicity.

The cross-sectional study uses the same Poisson regression framework but drops the value-added specification because there is a single time point. Again, because the admission counts in counties depend on the number of children living in the counties, we use the child population as an exposure variable in the Poisson model. Each county has two sets of observations: (1) White admissions and population and (2) Black admissions and population. Because the information about Black and White children is observed in the same county, the random effects model manages the non-independence. The other covariates in the cross-sectional model include urbanicity (based on the NCHS classification) and the index of social disadvantage.

An outline of the full model tested is shown as follows:

log(λ*_ij_*) = β_00__+_ β_10_ D*_ij_* + β_01_ C_BL*_j_*+ β_02_ C_WH*_j_* + β_11_ D*_ij_* C_BL*_j_* + β_12_ D*_ij_* C_WH*_j_* + β_03_ Social*_j_*+ β_13_ D*_ij_* Social*_j_* + β_04_ Urban*_j_* + β_14_ D*_ij_* Urban*_j_*+ Σα*_j_* + γ_0_*_j_* + γ_1_*_j_* D*_ij_* (4)

The term λ*_ij_* refers to the expected rate of child placements. β_00_ refers to the population-adjusted number of White placements (i.e., the event rate) and β_10_ measures the Black child/White Child placement rate difference. In other words, β_10_ is a direct measure of disparity. In essence, within the model, β_10_ considers the difference between each county’s Black child/White child placement rate. When the estimates of β_00_ and β_10_ are combined, the expected number of Black placements is obtained.

In the models, β_01_ and β_02_ show the impact of Black and White poverty rates on the expected number of placements. β_11_ and β_12_ represent the differential relationship between Black and White poverty rates with Black/White admission disparity.

## 6. Results

### 6.1. Child Poverty Rates and Poverty Rate Disparity

Table 1 shows how Black and White child poverty changed between 2000 and 2015 in the selected group of states. The average child poverty rates may be calculated with or without population weights. We present weighted poverty rates, which take county size into account.

The overall child poverty rate went up from 18% in 2000 to 22% in 2015. Poverty rates for Black children in the general population were consistently higher than those reported for White children. In fact, the difference is roughly three-fold (32% to 35% for Black children compared to 9% to 12% for White children). However, the disparity between Black child poverty and White child poverty went down over time (3.59 in 2000, 3.16 in 2010, and 2.98 in 2015). The decrease in disparity corresponded in part to the larger increase in White child poverty over the fifteen years.

Overall, when categorized by urbanicity, county poverty rates were the lowest in fringe counties and the highest in the noncore counties for both Black children and White children. Poverty rates in the remaining counties fell between the two poles. Though poverty rates for Black children are routinely higher, poverty rate disparity declined between 2000 and 2015 in all county classifications except the noncore counties.

### 6.2. Foster Care Admission Rates and Admission Rate Disparity

In Table 2 we translate the population and admission counts into their corresponding rates. As mentioned earlier, the admission rate is the number of children entering out-of-home care for every 1000 children in the underlying population by race. The admission disparity is the Black child admission rate divided by the White child admission rate. As with the child poverty rates, the analysis focuses on the weighted admission rates because they take county size into account.

The overall admission rates for the sample counties increased from 2.26 in 2000 to 2.42 in 2015. The increase was observed across county groups except in the large urban areas where admission rates declined from 2.83 in 2000 to 2.51 in 2015. The admission rates for Black children *declined* over time (from 4.87 in 2000 to 4.07 in 2015), while White child admission rates *increased* from 1.54 in 2000 to 1.87 in 2015. The drop in Black child placement rates from 2000 to 2015 was observed in large, fringe, and medium-sized counties. In smaller counties, the Black child placement rate trends were more up and down. For White children, in general, placement rates increased regardless of county size.

The disparity rates associated with these trends are found in the last three columns. The overall trend saw Black child/White child placement rate disparity go down between 2000 and 2015, from 3.17 to 2.17 in 2015. The overall decline in disparity, a product of a falling Black child placement rate and an increasing White child placement rate was about 30 percent. With that said, disparity persists, with the largest disparity rates found in large urban areas. As one moves away from the large urban areas, the Black child/White child differences become smaller.

### 6.3. Model Findings—Longitudinal Study

In the following section, we explore how the trends we observed in the descriptive analysis co-vary. We pay particular attention to race-specific child poverty rates as well as changes in those child poverty rates and the relationship of poverty rate changes with admission disparity. 

Model 1 in Table 3 includes the binary variable we use to measure the Black child/White child admission rate disparity (row 1). In Model 1, the intercept measures the White admission rate (the reference group) and row 1 measures the Black child/White child placement disparity. Model 1 also includes variables representing the effect of admission rates from prior years (row 2), a binary variable that shows the cohort effect (row 3), and the race-specific child poverty rates (rows 4 and 5). To capture the change in admissions and child poverty rates over time, two segments or cohorts—one from 2000 to 2010 and the other from 2010 to 2015—were formed from three data points. The cohort-specific binary variable captures the unique, between-cohort dynamics.

In Model 1 of Table 3, the Black/White disparity is 0.572 (row 1), a finding that is consistent with the idea that Black children are placed more frequently than White children after adjusting for the size of the population as shown in Table 2. Although the coefficient for prior admissions is small (0.000 in row 2), admissions are a continuous variable with a wide range. The significance associated with prior admissions suggests that counties with higher placement counts in the past tended to have higher placement counts in the future after adjusting for population size. The Cohort effect (Table 3, row 3) captures the time trend and shows the increase in the number of child placements as reported in Table 2. Regarding the effect of poverty on child placement, in counties with high White child poverty rates, after adjustment, child placements were higher (row 5); in counties with higher Black child poverty rates, child placements were just slightly elevated (row 4). The magnitude of the coefficients suggests that White child poverty has a more pronounced effect on child placement than Black child poverty.

Model 2 of Table 3 introduces changes in race-specific child poverty rates from time *t−*1 to time *t* (rows 8 and 9). In this model, the Black child/White child placement disparity persists (row 1) as does the effect of prior admissions (row 2). The time trend, however, turns negative (row 3) which suggests that child placements declined but for the increases in Black and White child poverty rates (rows 8 and 9). Compared with Model 1, the coefficient for White child poverty is much higher (4.408 compared to 1.274). 

The last model in Table 3—Model 3—adds the interactions of race-specific poverty rates with the binary variable that differentiates Black child placements from White child placements (rows 6 and 7). Compared with Models 1 and 2, the placement disparity in the model is much larger (1.084 compared to 0.572 and 0.566) because urbanicity is included in the model. As we saw in Table 2, the Black child/White child placement differences are larger in urban areas (row 12). The interaction of non-urban counties with Black/White disparity (row 13) suggests that the placement differences are lower in the counties outside of the large urban counties.

Once all of the parameters are estimated, the effect of prior admissions persists: counties with higher placement rates tended to have higher placement rates across all three time periods. The time trend, however, is now no longer significant (row 3), which indicates that the poverty rate changes reported in Table 1 accounted for the placement rate changes found in Table 2.

Regarding the relationship between race-specific poverty and placement rates, White child poverty has a substantial impact on child placement; Black child poverty has a relatively smaller impact (rows 4 and 5). The interaction terms in rows 6 and 7 show the differential relationship of race-specific poverty with placement disparity adjusted for population size. Black child poverty is associated with a relatively small increase in disparity. White child poverty, on the other hand, is associated with substantially reduced placement differences. Said differently, in counties with elevated rates of White child poverty, one would expect to find much smaller placement differences. In counties with elevated rates of Black child poverty, one would expect to find slightly higher placement differences.

The changes in Black child poverty rates had little effect on child placements (row 8) whereas the changes in White child poverty had a substantial effect (row 9). As for the relationship of changes in race-specific poverty with disparity, our findings (rows 10 and 11) are similar to what we observe in rows 6 and 7. The increase in Black child poverty was associated with slightly higher disparity; however, the increase in White poverty was associated with much smaller placement differences.

Model 3 incorporates urban classification to measure the influence of urban context on admissions and the derived admission disparity. It shows that, once adjusted for population size, admissions are more common in non-urban areas than in urban areas (row 12). Admission disparity was lower in non-urban areas than in urban areas as the negative coefficient of the interaction indicates (row 13). The impact size of the main effect (row 12) and the interaction effect (row 13) are similar but in opposite directions, indicating that the increase in admissions and the decline in admission disparity in non-urban counties are mainly driven by higher White child placement.

### 6.4. Model Findings—Cross-Sectional Study

For the cross-sectional study, we also constructed county random coefficients Poisson models with state fixed effects. Two differences are worth noting. First, as the sample of counties comes from a single period, we are no longer able to assess the change in placement rates relative to the change in poverty rates. Second, we add our measure of social disadvantage to the model. When considered alongside the poverty rate, the index is meant to capture the prevailing social conditions in a county in a more nuanced way.

The cross-sectional results are presented in Table 4. Model 1 investigates the Black/White placement disparity and the impact of Black and White poverty on placement frequency, net of population size differences. Model 2 additionally assesses the differential relationships between White and Black child poverty rates with Black/White admission disparity. Model 3 includes additional county variables: social disadvantage and urbanicity.

As in Table 3, the intercept references White child placements given the other parameters in the model and population size. The placement disparity (row 1) points to a higher number of placements for Black children in the years between 2017 and 2019. This finding is consistent with what we described longitudinally.

Regarding poverty, the model coefficients indicate that child placement counts are similar regardless of higher or lower Black child poverty rates (row 2). The effect associated with White child poverty shows higher child placements in counties with higher White child poverty rates (row 4). These race-specific effects of poverty on child placement are similar to the results found in the longitudinal study.

Model 2 shows the relationship between race-specific poverty rates and Black/White child admission disparity. It assesses whether counties with higher Black or White poverty rates have larger or smaller Black/White disparity. The coefficients for the interaction terms for both Black child poverty rates and White child poverty rates (rows 3 and 5) are both negative, indicating that counties with higher poverty rates have smaller Black/White admission disparity, regardless of whether one is talking about Black child or White child poverty. However, the magnitude of the coefficients is more pronounced in counties with elevated rates of White child poverty. Insofar as these results are similar to those reported from the longitudinal study, these findings reinforce the idea that to understand the persistent nature of disparity, one must understand the distinct effects of Black and White child poverty side by side.

Model 3 incorporates the social disadvantage index and urbanicity variable in addition to Black and White child poverty rates. Consequently, the intercept in Model 3 is related to White child admissions in urban counties with high levels of social disadvantage. The negative coefficient (−0.288) suggests that White child placements are low in urban counties with high levels of social disadvantage. This is likely because urban areas have relatively low rates of White child poverty but high rates of Black child poverty that are correlated with our measure of social disadvantage (which is not race specific). Taken together, the results show that White child placements are low in urban counties. At the same time, the placement disparity (0.802—row 1) is substantial.

The relatively low number of White child placements in urban areas explains why counties rated as having moderate levels of social disadvantage have higher White child placements than urban counties (row 8). Ordinarily, one would expect to find higher placements in counties with higher levels of social disadvantage. However, the contrast is with urban counties where social disadvantage is high but White poverty is low. In this case, the increase in White placement in counties with moderate levels of social disadvantages reflects the unique population composition one finds when comparing urban areas with non-urban areas. Poverty rates for White children are higher in the outlying areas. Finally, the results point to higher placement disparity as one moves along the social disadvantage index from high to low, with placement disparity at its highest level in the counties with low levels of social disadvantage. This last point, aligned as it is with our findings that show lower disparity in high-poverty areas, stands as an additional counterpoint to claims that placement disparity is a by-product of Black child poverty rates only [51,52].

## 7. Limitations

Our study has several limitations, and it is important to acknowledge what they are. First, we have omitted from our study all the other decision points along the service continuum where differential treatment of White and Black children is possible: reporting, investigation, substantiation, and termination of parental rights, among them. We also left out analysis based on the reason for entry. Obviously, findings in all these areas are critical to our overall understanding of disparity. Nevertheless, we were interested in a fundamental question: how often in this collection of counties and states is someone being asked to raise someone else’s children, even if only for a short time? Here in the U.S. and elsewhere around the world, parents have the responsibility of raising their children. The frequency with which the parent/child living arrangement comes undone with a placement in foster care is an important indicator of just how well the community at large supports parents in this fundamental endeavor.

Second, we only examined the experience of Black children and White children even though America is far more diverse. Other studies indicate that rates of placement for Hispanics/Latinx children follow yet a third trajectory over time [40]. Other racial and ethnic groups, including Asians and Native Americans, are, have been, or should be the subject of studies that examine differential treatment. We limited our study to Black children and White children because of the challenges we faced creating a longitudinal file of race-specific population characteristics at the county level. Much of what is of interest today was not being collected in 2000. We have no doubt that the analytical strategy applied here would yield insights regarding the treatment of those children by child protection systems with relevance to the groups themselves *and* how we understand the treatment of Black and White children. In this context, we also fully acknowledge that categories such as Black, White, Hispanic, Asian, and Native American are imprecise. That fact is one reason we remain circumspect with regard to what we have found and what it means.

Third, counties are hardly a proxy for communities. If we were to disaggregate county data and consider smaller spatial scales, as Coulton and her colleagues [53] did, we might find something altogether different, something even more nuanced. That said, we do have the advantage of observing counties across multiple jurisdictions over multiple years. As expected, the comparative perspective adds considerably to the body of findings in ways that are not possible within a single jurisdiction.

Fourth, although we were able to include measures of the county’s social and economic context in addition to poverty for the cross-sectional study, we were unable to locate race-specific measures of social disadvantage (see Table A5) using consistent definitions. Future efforts to assess the social context should continue to explore approaches to measuring the impact of the broader socio-economic context both cross-sectionally and longitudinally.

Finally, social structural variation is hardly the only way in which counties vary from one another. Whether at the state or local level, child welfare *systems* differ considerably in ways that were not measured here. For example, state investments in child protection programs vary considerably. Given that resource allocation is, theoretically, one of the ways in which structural bias plays out in operational contexts, the omission of system resources and other measures of system structure is anything but a trivial matter. We are unlikely to understand why placement rates and poverty rates are so different for Black and White children without also understanding the allocation of resources as one of the ways bias manifests. We take solace in the fact that our study raises this important question.

## 8. Discussion

In this study and others [3,20,54,55], we have tried to deepen what we know about placement disparity by studying placement and poverty with specific attention paid to the experience of Black children and youth in contrast to the experience of White children and youth. This approach, which is consistent with the approach taken by other scholars [32,33,53,56,57,58,59,60,61], emphasizes the unique context in which Black children live out their lives. To capture the distinctive features of the Black experience with placement systems, we isolated Black placement and Black child poverty from White placement and White child poverty. Doing so reveals a sharp and important contrast.

To carry out the study, we linked two observations in a longitudinal study of disparity: rising poverty rates in non-urban counties from 2000 to 2015 on the one hand and rising placement rates in non-urban counties on the other. Given the connection between poverty and placement *and* the Black/White differences in where people live along the urban/rural continuum, we were interested in how our understanding of disparity is informed by the underlying poverty/population dynamic. We also examined disparity at a particular point in time using additional measures of the social and socioeconomic characteristics of counties in which children live.

As a general matter, the study confirms the fact that placement is more common among Black children and youth when compared with those reported for White children and youth. If, however, we consider the relationship between poverty and placement, the narrative quickly becomes more complicated, especially as we look over time. Higher child poverty is associated with more frequent placement, but race matters. Black child poverty is associated with higher Black child placement, but not to the same extent as the association between White child poverty and White child placement. As White poverty rates increased between 2000 and 2015, so too did placement for both Black and White children. Placement for White and Black children living in the midst of Black poverty increased as well, but the changes were much smaller. As a consequence, in counties where the placements for White children were rising because of White child poverty, the placement gap narrowed. Including additional measures of county context (i.e., social disadvantage) in the cross-sectional study did not change the narrative that disparity is lower in counties where poverty is greater.

## 9. Conclusions

From this set of findings, a persistent conclusion emerges. The magnitude of Black/White placement differences is to a very large extent conditional on where one looks and when one looks. For that reason, it is unlikely that a single explanation accounts for the disparity we want to change. This is not meant to suggest that common explanations for disparity—structural bias, racism, and social and economic inequality—are not applicable. Rather, the findings here suggest that sweeping, one-size-fits-all generalizations do little to push the search for solutions very far. In the United States, the history of differential treatment of Blacks by the White society includes examples of services being withheld (e.g., education) and examples of heightened scrutiny (e.g., policing). We ought to consider systematically whether one or the other or both possibilities account for placement disparities. For example, in counties with rising White poverty rates, what does access to the child welfare system look like for Black families?

Regardless of race and ethnicity, poverty and its correlates do not make holding a family together easier in contemporary society. For researchers and policymakers, the challenge rests first with understanding the mediating and moderating factors that mitigate the corrosive influence of poverty on family life and then with understanding the differential allocation of those factors by race and ethnicity. Only then will we know how to strengthen families and the communities where they live. To that end, studies that further examine the variation in placement disparity are essential.

## Figures and Tables

**Table 1:** Child poverty rates and poverty rate disparity in the general population by race and year.

	Overall Child Poverty			Black Poverty			White Poverty			Disparity		
Urbanicity	2000	2010	2015	2000	2010	2015	2000	2010	2015	2000	2010	2015
Overall	18%	20%	22%	32%	33%	35%	9%	11%	12%	3.59	3.16	2.98
Large	20%	21%	24%	33%	33%	35%	8%	8%	10%	4.31	3.92	3.55
Fringe	10%	12%	14%	23%	24%	25%	6%	7%	8%	3.97	3.36	3.00
Medium	19%	22%	25%	33%	36%	39%	9%	11%	13%	3.50	3.19	2.90
Small	18%	21%	23%	36%	40%	40%	11%	14%	15%	3.23	2.95	2.70
Micro	19%	24%	26%	39%	47%	49%	14%	18%	20%	2.67	2.67	2.47
Noncore	22%	26%	28%	38%	47%	48%	18%	20%	22%	2.16	2.30	2.17

**Table 2:** Foster care admission rates and admission rate disparity by race and year.

	Overall Admission Rate			Black Admission Rate			White Admission Rate			Disparity		
Urbanicity	2000	2010	2015	2000	2010	2015	2000	2010	2015	2000	2010	2015
Overall	2.26	2.31	2.42	4.87	4.09	4.07	1.54	1.75	1.87	3.17	2.34	2.17
Large	2.83	2.55	2.51	5.41	4.55	4.25	1.51	1.43	1.54	3.59	3.18	2.76
Fringe	1.37	1.45	1.51	3.44	2.93	2.95	1.02	1.14	1.19	3.37	2.57	2.47
Medium	2.44	2.66	2.88	5.18	4.18	4.83	1.89	2.26	2.34	2.74	1.85	2.07
Small	2.27	2.91	3.22	4.54	5.10	5.38	1.93	2.50	2.79	2.35	2.04	1.93
Micro	2.19	2.68	2.96	3.67	3.15	3.60	2.04	2.60	2.84	1.80	1.21	1.27
Noncore	1.98	2.48	3.04	2.90	3.24	2.91	1.83	2.32	3.08	1.59	1.40	0.94

**Table 3:** Parameter Estimates for Admissions Controlling for Race-Specific Rates of Child Poverty, Changes in Race-Specific Child Poverty Rates, Historical Placements, and Urbanicity.

	Model 1			Model 2			Model 3		
Parameter	Coeff.	St. Err.	Pr. > |t|	Coeff.	St. Err.	Pr. > |t|	Coeff.	St. Err.	Pr. > |t|
Intercept	0.665	0.094	<0.001	0.269	0.089	0.003	0.016	0.089	0.861
1. Disparity ^1^	0.572	0.032	<0.001	0.566	0.032	<0.001	1.084	0.075	<0.001
2. Prior admission	0.000	0.000	<0.001	0.000	0.000	<0.001	0.000	0.000	<0.001
3. Cohort	0.033	0.007	<0.001	−0.025	0.008	0.001	−0.006	0.008	0.433
4. Black child poverty	0.202	0.054	0.000	0.286	0.095	0.003	0.184	0.096	0.056
5. White child poverty	1.274	0.208	<0.001	4.408	0.278	<0.001	4.049	0.296	<0.001
6. Disparity * Black child poverty ^2^							0.544	0.192	0.005
7. Disparity * White child poverty							−2.536	0.441	<0.001
8. Changes in Black child poverty				0.146	0.059	0.013	0.075	0.061	0.219
9. Changes in White child poverty				2.989	0.187	<0.001	2.833	0.206	<0.001
10. Disparity * Changes in Black child poverty							0.357	0.134	0.008
11. Disparity * Changes in White child poverty							−1.409	0.347	<0.001
12. Non-urban							0.461	0.054	<0.001
13. Disparity * Non-urban							−0.470	0.072	<0.001

^1^ This is a binary variable to differentiate the observations (the records) of Black children from the observations (the records) of White children. We refer to this binary variable as Disparity for interpretative clarity. By definition, the coefficient in row 1 represents the average placement rate difference observed when each county’s Black child placement rate is compared with each county’s White child placement rate). ^2^ Asterisks in this table indicate the use of an interaction term as the parameter.

**Table 4:** Parameter Estimates for Admissions Controlling for Race-Specific Child Poverty Rates, an Index of Social Disadvantage, and Urbanicity: 2017–2019 Combined.

	Model 1			Model 2			Model 3		
Parameter	Coeff.	St. Err.	Pr. > |t|	Coeff.	St. Err.	Pr. > |t|	Coeff.	St. Err.	Pr. > |t|
Intercept	0.216	0.101	0.033	0.181	0.101	0.073	−0.288	0.133	0.031
1. Disparity ^1^	0.631	0.029	<0.001	1.437	0.069	<0.001	0.802	0.130	<0.001
2. Black Child Poverty	0.000	0.001	0.940	0.000	0.001	0.722	−0.001	0.001	0.608
3. Disparity * Black Child Poverty ^2^				−0.008	0.001	<0.001	−0.004	0.001	0.003
4. White Child Poverty	0.046	0.003	<0.001	0.047	0.003	<0.001	0.038	0.003	<0.001
5. Disparity * White Child Poverty				−0.032	0.003	<0.001	−0.023	0.004	<0.001
6. Non-urban							0.473	0.056	<0.001
7. Disparity * Non-urban							−0.160	0.068	0.019
8. Soc Disadvantage—med							0.317	0.082	0.000
9. Soc Disadvantage—low							0.002	0.126	0.989
10. Disparity * Soc Disadvantage—med							0.511	0.093	<0.001
11. Disparity * Soc Disadvantage—low							0.957	0.155	<0.001

^1^ This is a binary variable to differentiate the observations (the records) of Black children from the observations (the records) of White children. We refer to this binary variable as Disparity for interpretative clarity. By definition, the coefficient in row 1 represents the average placement rate difference observed when each county’s Black child placement rate is compared with each county’s White child placement rate). ^2^ Asterisks in this table indicate the use of an interaction term as the parameter.

## Data Availability

Restrictions apply to the availability of these data. Data were obtained from members of the Center for State Child Welfare Data and are available from the authors with the permission of Data Center member states.

## Appendix

### Appendix A.1. Study Sample

One key to understanding placement disparity is tied up with where Black and White children live along the urban/rural continuum. Table A1 and Table A2, which show the count of children for both population and foster care placement for the longitudinal study, illustrate why this is important. Briefly, between 2000 and 2015, county demographics shifted significantly as Black children and their families left urban counties for the counties further from the urban core. The same can be said for foster care placement.

We show these trends in two ways: row percentages (Table A1) show the percentage of children in each county group who are White and Black; the column percentages (Table A2), show where White and Black children live across the county groups.

Similarly, Table A3 and Table A4 present the child population and foster care admissions for the cross-sectional study.

**Table A1:** Longitudinal Study Sample—Row Percentages.

	2000		2010		2015	
	Black Children	White Children	Black Children	White Children	Black Children	White Children
Child Population						
Large	3,079,302	5,980,889	2,839,039	5,080,614	2,778,010	5,007,792
Fringe	899,281	5,356,061	1,045,859	5,053,536	1,040,015	4,756,699
Medium	720,392	3,549,694	876,722	3,284,212	871,269	3,117,186
Small	244,770	1,620,112	280,760	1,489,078	277,237	1,374,044
Micro	157,579	1,576,299	278,138	1,539,825	269,548	1,361,205
Noncore	161,940	967,244	225,234	1,060,961	204,677	707,772
Total	5,263,264	19,050,299	5,545,752	17,508,226	5,440,756	16,324,698
Large	34%	66%	36%	64%	36%	64%
Fringe	14%	86%	17%	83%	18%	82%
Medium	17%	83%	21%	79%	22%	78%
Small	13%	87%	16%	84%	17%	83%
Micro	9%	91%	15%	85%	17%	83%
Noncore	14%	86%	18%	82%	22%	78%
Total	22%	78%	24%	76%	25%	75%
Foster Care Admissions						
Large	16,648	9010	12,925	7276	11,819	7722
Fringe	3091	5465	3065	5763	3066	5683
Medium	3729	6694	3661	7423	4206	7279
Small	1112	3127	1431	3716	1491	3827
Micro	579	3214	877	3999	970	3865
Noncore	470	1769	730	2463	596	2182
Total	25,629	29,279	22,689	30,640	22,148	30,558
Large	65%	35%	64%	36%	60%	40%
Fringe	36%	64%	35%	65%	35%	65%
Medium	36%	64%	33%	67%	37%	63%
Small	26%	74%	28%	72%	28%	72%
Micro	15%	85%	18%	82%	20%	80%
Noncore	21%	79%	23%	77%	21%	79%
Total	47%	53%	43%	57%	42%	58%

**Table A2:** Longitudinal Study Sample—Column Percentages.

	Black Children			White Children		
	2000	2010	2015	2000	2010	2015
Child Population						
Large	3,079,302	2,839,039	2,778,010	5,980,889	5,080,614	5,007,792
Fringe	899,281	1,045,859	1,040,015	5,356,061	5,053,536	4,756,699
Medium	720,392	876,722	871,269	3,549,694	3,284,212	3,117,186
Small	244,770	280,760	277,237	1,620,112	1,489,078	1,374,044
Micro	157,579	278,138	269,548	1,576,299	1,539,825	1,361,205
Noncore	161,940	225,234	204,677	967,244	1,060,961	707,772
Total	5,263,264	5,545,752	5,440,756	19,050,299	17,508,226	16,324,698
Large	59%	51%	51%	31%	29%	31%
Fringe	17%	19%	19%	28%	29%	29%
Medium	14%	16%	16%	19%	19%	19%
Small	5%	5%	5%	9%	9%	8%
Micro	3%	5%	5%	8%	9%	8%
Noncore	3%	4%	4%	5%	6%	4%
Total	100%	100%	100%	100%	100%	100%
Foster Care Admissions						
Large	16,648	12,925	11,819	9010	7276	7722
Fringe	3091	3065	3066	5465	5763	5683
Medium	3729	3661	4206	6694	7423	7279
Small	1112	1431	1491	3127	3716	3827
Micro	579	877	970	3214	3999	3865
Noncore	470	730	596	1769	2463	2182
Total	25,629	22,689	22,148	29,279	30,640	30,558
Large	65%	57%	53%	31%	24%	25%
Fringe	12%	14%	14%	19%	19%	19%
Medium	15%	16%	19%	23%	24%	24%
Small	4%	6%	7%	11%	12%	13%
Micro	2%	4%	4%	11%	13%	13%
Noncore	2%	3%	3%	6%	8%	7%
Total	100%	100%	100%	100%	100%	100%

**Table A3:** Cross-Sectional Study Sample—Row Percentages.

	Black Children	White Children
Child Population *		
Large	8,334,030	15,023,376
Fringe	3,125,034	14,538,069
Medium	2,616,630	9,566,145
Small	836,007	4,362,612
Micro	815,808	4,477,377
Noncore	633,219	3,507,315
Total	16,360,728	51,474,894
Large	36%	64%
Fringe	18%	82%
Medium	21%	79%
Small	16%	84%
Micro	15%	85%
Noncore	15%	85%
Total	24%	76%
Foster Care Admissions *		
Large	34,533	20,216
Fringe	9105	18,478
Medium	13,354	24,549
Small	5239	13,416
Micro	3704	16,595
Noncore	2301	14,071
Total	68,236	107,325
Large	63%	37%
Fringe	33%	67%
Medium	35%	65%
Small	28%	72%
Micro	18%	82%
Noncore	14%	86%
Total	39%	61%

* The population estimates are from the 2011–2015 American Community Survey multiplied by three. The foster care admissions are from the years 2017–2019, combined.

**Table A4:** Cross-Sectional Study Sample—Column Percentages.

	Black Children	White Children
Child Population *		
Large	8,334,030	15,023,376
Fringe	3,125,034	14,538,069
Medium	2,616,630	9,566,145
Small	836,007	4,362,612
Micro	815,808	4,477,377
Noncore	633,219	3,507,315
Total	16,360,728	51,474,894
Large	51%	29%
Fringe	19%	28%
Medium	16%	19%
Small	5%	8%
Micro	5%	9%
Noncore	4%	7%
Total	100%	100%
Foster Care Admissions *		
Large	34,533	20,216
Fringe	9105	18,478
Medium	13,354	24,549
Small	5239	13,416
Micro	3704	16,595
Noncore	2301	14,071
Total	68,236	107,325
Large	51%	19%
Fringe	13%	17%
Medium	20%	23%
Small	8%	13%
Micro	5%	15%
Noncore	3%	13%
Total	100%	100%

* The population estimates are from the 2011–2015 American Community Survey multiplied by three. The foster care admissions are from the years 2017–2019, combined.

### Appendix A.2. Social Disadvantage Index

To create the social disadvantage index, we took the following steps for each of the 20 indicators listed in Table A5 below:

First, for each of the 20 indicators, we calculated each county’s standard deviation from the average of all of the counties in the study sample. Counties were assigned a value of −1 for each indicator for which the county was more than 1.5 standard deviations *above* the mean (these are counties with a *higher* than average percentage of people needing support according to that indicator). Conversely, counties were assigned a value of 1 for each indicator for which the county was more than one standard deviation *below* the mean (these are counties with a *lower* than average percentage of people needing support according to that indicator). All other counties were assigned a value of 0.

Next, we summed these values (−1, 0, 1) across all 20 variables to produce a total score for each county. Theoretically, the final scores range from −20 to +20. Finally, the counties were grouped into seven categories based on the summed score, where counties in category 7 were those with the most favorable ecological circumstances for raising children, and counties in category 1 were those where parents would face far more challenging circumstances when raising children.

**Table A5:** County-Level Indicators Included in the Social Disadvantage Index.

Domain	County-Level Indicator	Definition	Data Source	Year
Family structure	Female-headed households	Percentage of female-headed households, overall and by race (Black/White)	American Community Survey	5-year estimates for calendar years 2011–2015
	Children in single parent families	Percentage of children that live in a household headed by single parent	American Community Survey	5-year estimates for calendar years 2016–2020
Poverty	Children in poverty	Percentage of children under age 18 with poverty status determined who were below the poverty line in the past year, overall and by race (Black/White)	American Community Survey	5-year estimates for calendar years 2011–2015
	Families eligible for public assistance	Percentage of families eligible for public assistance	American Community Survey	5-year estimates for calendar years 2014–2018
	Free/reduced price lunch	Percentage of children eligible for free or reduced-price lunch	National Center for Education Statistics	2020
	Children covered by Medicaid	Percentage of under 19 population covered by Medicaid	American Community Survey	5-year estimates for calendar years 2014–2018
	Income ratio	Income ratio (80th percentile to 20th percentile)	American Community Survey	5-year estimates for calendar years 2016–2020
General social indicators	Low birth weight	Percentage of live births with low birthweight (2500 g)	National Center for Health Statistics	2020
	Child mortality	Number of deaths among children under age 18 per 100,000 population	National Center for Health Statistics	2015
	Infant mortality	Infant mortality rates refer to the number of deaths under age 1 year per 1000 live births	National Center for Health Statistics	2020
	Teen births	Number of births per 1000 female population ages 15–19	National Center for Health Statistics	2020
	Drug deaths	Number of drug poisoning deaths per 100,000 population	National Center for Health Statistics	2020
	Excessive drinking	Percentage of adults reporting binge or heavy drinking	Behavioral Risk Factor Surveillance System	2020
	Uninsured children	Percentage of children under age 19 without health insurance	Small Area Health Insurance Estimates	2020
	High housing cost	Percentage of households with high housing costs	Comprehensive Housing Affordability Strategy	2020
	Housing problems	Percentage of households with at least 1 of 4 housing problems: overcrowding, high housing costs, lack of kitchen facilities, or lack of plumbing facilities	Comprehensive Housing Affordability Strategy	2020
